# Comparison of Stoma Formation Using Circular Stapler Fascial-Aperture Technique (Stapled Trephination) Versus the Traditional Method (Cruciate Fascial Incision): A Retrospective Study at King Faisal Specialist Hospital

**DOI:** 10.7759/cureus.97648

**Published:** 2025-11-24

**Authors:** Khaled Afasha, Yasir A Alharbi, Alshaymaa Safarji, Jood Albehani, Saffanah AlAhmadi, Hani A Redwan

**Affiliations:** 1 General Surgery, King Faisal Specialist Hospital and Research Centre, Madinah, SAU; 2 General Surgery, King Fahad General Hospital, Madinah, SAU; 3 Clinical Sciences, Al-Rayan National College of Medicine, Al-Rayan National Colleges, Madinah, SAU

**Keywords:** circular-stapler fascial-aperture technique (stapled trephination), complications, retrospective study, risk factors, stoma formation, traditional method (cruciate fascial incision)

## Abstract

Background and aim: Stoma formation is a critical component of colorectal surgery, with postoperative complications, such as parastomal hernia (PSH), remaining common. The traditional cruciate fascial incision has been the conventional approach, whereas the circular-stapler fascial-aperture technique (stapled trephination) has emerged as an alternative, potentially influencing complication rates. This study aimed to compare two fascial-aperture techniques-traditional cruciate incision and circular-stapler (stapled trephination) and evaluate complication rates and patient-related factors (age, gender, body mass index (BMI), and type of surgery.

Materials and methods: A retrospective hospital-based study was conducted at King Faisal Specialist Hospital, Madinah, Saudi Arabia, from 2021 to 2025. A total of 46 patients undergoing elective gastrointestinal surgeries with stoma formation were included (37 traditional; nine end-to-end anastomosis (EEA)). Patient demographics, types of surgery, and postoperative complications were collected from hospital records. Data were analyzed using IBM SPSS Statistics for Windows, version 20 (IBM Corp., Armonk, New York, United States), with chi-square and Fisher's exact tests applied as appropriate.

Results: The study sample consisted of 46 participants with a mean age of 52.1 years. The majority were male (n=34, 73.9%), and 26 patients had a BMI greater than 25 kg/m^2^ (56.5%). The stoma type was mostly traditional (n=37, 80.4%) versus EEA (n=9, 19.6%). Complications were observed in 13 patients (28.3%), with a higher proportion in the EEA (n=3, 33.3%) than in the traditional group (n=10, 27.0%). The most common complication was parastomal hernia (PSH) (17.4%), which occurred more frequently in the traditional group (18.9%) than in the EEA group (11.1%). Less frequent complications included partial necrosis (2.2%) and ischemia (2.2%) in the EEA group, and ileus (2.2%) and abscesses (4.3%) in the traditional group. No significant associations were found between demographic characteristics (age, gender, and BMI) or surgery types and complications (all p > 0.05).

Conclusions: Both traditional and EEA methods of stoma formation demonstrated insignificant complication rates, with each technique exhibiting distinct complication profiles. The most common complication was PSH, which occurred more frequently in the traditional than in the EEA group. Larger, multicenter prospective studies with longer follow-up are needed to determine the long-term safety and efficacy of these methods and to guide surgical decision-making.

## Introduction

The establishment of a stoma plays a central role in colorectal surgery, requiring meticulous surgical care to minimize postoperative complications. Various classical techniques, such as the cruciate fascial incision, have been extensively utilized in stoma formation. However, the introduction of new surgical instruments, such as circular staplers, offers potential benefits in terms of efficiency and complication rates [1].

Parastomal hernia (PSH) is a type of incisional hernia (IH) that occurs when abdominal contents protrude through a defect in the stoma's abdominal wall. The incidence of PSH, a painful complication of stoma creation, varies widely, ranging from 30% to 60% depending on stoma type and follow-up period [2,3]. In addition to reducing patients' quality of life, PSH may cause pain, fecal incontinence, inconsistent stoma appliance use, and skin inflammation [4,5]. Moreover, PSH can lead to serious, potentially fatal complications such as obstruction, necrosis, or perforation [6,7]. Despite their prevalence, these hernias remain difficult to manage.

Several herniation prevention techniques have been documented; however, none have consistently reduced the incidence of PSH. An in vitro experimental simulation study demonstrated that the conventional cruciate incision method generates higher pressure at the ostomy site than the circular excision method, suggesting that the traditional approach may pose a greater risk of PSH. Conversely, the circular excision method may provide a more uniform pressure distribution, thereby reducing the occurrence of PSH [8]. Moreover, the Stapled Mesh Stoma Reinforcement Technique (SMART) has been proposed as a preventive measure against PSH. This procedure involves inserting a circular polypropylene mesh during stoma creation to reinforce the abdominal wall and prevent herniation. Although early results indicate a decreasing trend in PSH incidence, the long-term outcomes remain to be fully established [1]. A recent randomized trial [9] comparing preventive mesh placement, circular sheath incision, and cruciate sheath incision reported no difference in PSH rates. Previous studies have shown favorable short-term outcomes with the use of a circular stapler (end-to-end anastomosis (EEA)) for creating the stoma aperture [10,11].

Nevertheless, these studies differed in inclusion criteria, methodology, PSH evaluation, and terminology. Another study prospectively assessed the safety of using a stapling device to create stomal apertures in end colostomies, with a primary focus on the 30-day rate of stoma-related complications [12]. Literature has reported early complication rates of approximately 34%, with values ranging from 25% to 80% [13]. Kazi et al. established an unacceptable complication rate at 45% for the upper limit of the 95% CI [12].

This study aimed to determine the incidence of complications in traditional and EEA stoma procedures and to assess the associations between patient characteristics (e.g., BMI, age, and gender), types of surgery, and complication rates.

## Materials and methods

This hospital-based retrospective study was conducted in the Department of General Surgery at King Faisal Specialist Hospital, Madinah, Saudi Arabia, from 2021 to 2025. Patient confidentiality and data protection were strictly maintained.

Study population

The study population comprised all patients who underwent elective gastrointestinal surgeries. Patients were categorized into two groups based on the type of anastomosis: (i) traditional cruciate fascial incision and (ii) circular stapler (EEA).

Inclusion Criteria

All patients admitted to the Department of General Surgery at King Faisal Specialist Hospital, Madinah, Saudi Arabia, who required elective gastrointestinal surgeries and underwent bowel anastomosis using either the traditional cruciate fascial incision or the circular stapler (EEA) for various benign and malignant conditions were included. Both genders aged > 12 years who underwent gastrointestinal surgeries were eligible.

Exclusion Criteria

Patients aged ≤12 years; those undergoing gastrointestinal anastomosis in emergency settings, pregnant women, patients receiving radiotherapy, patients with coagulopathy or on anticoagulant therapy, patients who underwent other types of anastomosis, and those whose anastomosis was performed in other hospitals were excluded.

All patients who met the inclusion criteria during the study period were included; thus, a total of 46 patients (37 using the traditional method and nine using the EEA method) were included.

Surgery process and materials

In the traditional cruciate fascial incision group, the suture material and anastomosis technique were determined according to the individual surgeon's preference. In the stapler group, anastomosis was performed using circular anastomosing staplers. Circular stapler fascial-aperture technique was performed using EEA circular staplers (sizes 25 mm and 28 mm; Ethicon, Inc., Raritan, New Jersey, United States), each device being new for every procedure. The stapler was introduced through the fascial layer with the anvil positioned centrally, excising the fascial disk in a single firing under counter-pressure.

Data collection and outcomes

Data were extracted from electronic patient records and organized into an Excel sheet containing information on gender, age, BMI, type of stoma, type of surgery, and type of complications. The primary outcome is explicitly stated as “any stoma-related complication within 30 days post-operation,” and PSH as the secondary outcome, assessed at ≥ 6 months of follow-up.

Data analysis

Data analysis was performed using the IBM SPSS Statistics for Windows, version 20 (IBM Corp., Armonk, New York, United States). Descriptive analysis was conducted for baseline patient characteristics (age, gender, and BMI). Complication rates between EEA and traditional methods were compared using Pearson's Chi-square or Fisher's exact test, as appropriate. Comparisons between the two groups were performed to identify predictors of complications. A p-value of less than 0.050 was considered statistically significant.

## Results

The study sample consisted of 46 participants with a mean age of 52.1 years, ranging from 17 to 97 years, indicating the inclusion of both young and elderly patients. Males (34, 73.9%) significantly outnumbered females (12, 26.1%) (p < 0.001). More than half of the participants (26, 56.5%) had a BMI greater than 25, while 20 patients (43.5%) had a BMI ≤ 25 (p = 0.238), suggesting that overweight or obesity was common among the study group. Regarding stoma type, 37 patients underwent the traditional method, which was markedly more frequent (80.4%) than the nine patients who underwent the EEA method (19.6%) (p < 0.0001), indicating a clear predominance of traditional stoma formation in this study (Table 1).

**Table 1:** Demographic characteristics and type of stoma in all participants (N = 46) EEA: end-to-end anastomosis

Characteristics	Value	Chi-Square	P-value
Age (years), mean±SD, range)	52.13±20.26 (17-97)		
Gender, n (%)		10.522	0.001
Male	34 (73.9%)		
Female	12 (26.1%)		
BMI (kg/m^2^), n (%)		1.391	0.238
≤25	20 (43.5%)		
>25	26 (56.5%)		
Type of Stoma, n (%)		17.043	0.0001
EEA	9 (19.6%)		
Traditional	37 (80.4%)		

**Figure 1:**
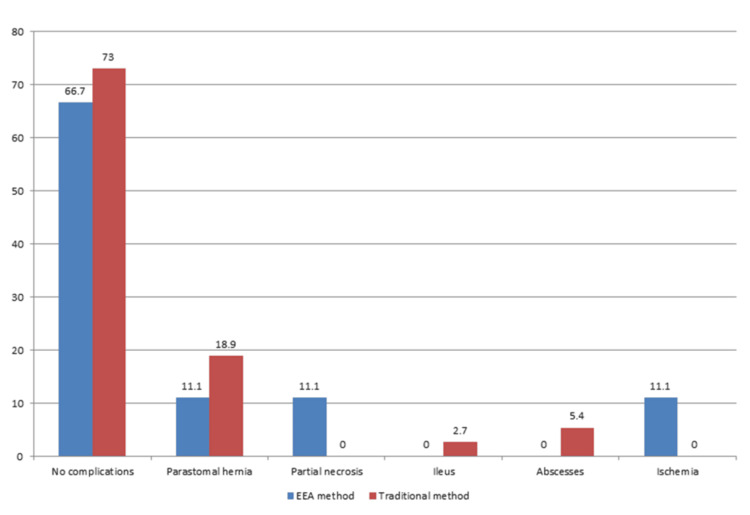
Complications recorded according to type of stoma

Overall, complications were observed in 13 patients (28.3%), with a slightly higher proportion in the EEA group (3, 33.3%) compared to the traditional group (10, 27.0%); however, this difference was not statistically significant (p = 0.698). The most common complication was a parastomal hernia, reported in eight patients (17.4%), which occurred more frequently in the traditional group (7, 18.9%) than in the EEA group (1, 11.1%). Less frequent complications included partial necrosis (1, 2.2%), ileus (1, 2.2%), abscesses (2, 4.3%), and ischemia (1, 2.2%). Notably, partial necrosis and ischemia were observed only in the EEA group, whereas ileus and abscesses occurred exclusively in the traditional group. None of these differences reached statistical significance (p = 0.181) (Table 2 and Figure 1).

**Table 2:** Complications recorded according to type of stoma

Complications	All patients (n= 46), n (%)	EEA (n=9), n (%)	Traditional (n=37), n (%)	Chi-Square	P-value
Presence of complications				0.142	0.698
No complications	33 (71.7%)	6 (66.7%)	27 (73.0%)		
Complications	13 (28.3%)	3 (33.3%)	10 (27.0%)		
Types of complications				7.262	0.181
Parastomal hernia	8 (17.4%)	1 (11.1%)	7 (18.9%)		
Partial necrosis	1 (2.2%)	1 (11.1%)	-		
Ileus	1 (2.2%)	-	1 (2.7%)		
Abscesses	2 (4.3%)	-	2 (5.4%)		
Ischemia	1 (2.2%)	1 (11.1%)	-		

No significant associations were found between demographic characteristics (age, gender, BMI) and the occurrence of complications (all p > 0.05). Complications appeared across all age groups except in patients younger than 25 years, with PSH being most frequent in the age group of 51-75 years (n=4, 8.7%). Both male and female patients experienced complications, but with a similar distribution (p = 0.822). Regarding BMI, patients with BMI > 25 showed a higher proportion of complications, particularly PSH (n=6, 13.0%) and other events such as partial necrosis (n=1, 2.2%) and ileus (n=1, 2.2%), while patients with BMI ≤ 25 had fewer complications overall, such as PSH (n=2, 4.3%), abscess (n=1, 2.2%), and ischemia (n=1, 2.2%). Although these trends suggest overweight/obesity may increase the likelihood of complications, the differences were not statistically significant (p = 0.097) (Table 3).

**Table 3:** Complications recorded according to demographic characteristics of patients. Note: Percentage from total number of patients

Characteristics	No complications (n=33)	Parastomal hernia (n=9)	Partial necrosis (n=1)	Ileus (n=1)	Abscesses (n=2)	Ischemia (n=1)	Chi-Square	P-value
Age (years)							11.290	0.952
< 25 (n=5)	5 (10.9%)	-	-	-	-			
26-50 (n=16)	11 (23.9%)	2 (4.3%)	1 (2.2%)	-	1 (2.2%)			
51-75 (n=18)	12 (26.1%)	4 (8.7%)	-	1 (2.2%)	1 (2.2%)			
>75 (n=7)	5 (10.9%)	2 (4.3%)	-	-	-			
Gender							3.040	0.822
Male (n=34)	25 (54.3%)	6 (10.9%)	1 (2.2%)	1 (2.2%)	1 (2.2%)			
Female (n=12)	8 (17.4%)	3 (6.5%)	-	-	1 (2.2%)			
BMI (kg/m^2^)							7.353	0.097
≤25 (n=27)	22 (47.8%)	2 (4.3%)	-	-	1 (2.2%)	1 (2.2%)		
>25 (n=19)	11 (23.9%)	6 (13.0%)	1 (2.2%)	1 (2.2%)	-	-		

Overall, complications were observed in 13 (28.3%) patients, with a slightly higher proportion in the EEA group (n=3, 33.3%) compared to the traditional group (n=10, 27.0%); however, this difference was not statistically significant (p = 0.698). Complications occurred across different types of surgery but without statistically significant differences (p = 0.720). The highest frequency of complications was noted after laparoscopic loop ileostomy and laparoscopic loop end colostomy, with parastomal hernia being the most common event. Rare complications such as 1 partial necrosis (2.2%) and 1 ischemia (2.2%) were seen following laparotomy and laparoscopic loop end colostomy, respectively. In contrast, patients undergoing open anterior resection with end colostomy and surgery for semi-obstructing sigmoid adenocarcinoma had no recorded complications. Overall, complication rates appeared more variable in laparoscopic procedures, but these differences did not reach statistical significance (Table 4).

**Table 4:** Complications recorded according to types of surgery done. Note: Percentage from total number of patients.

Types of surgery	No complications (n=33)	Parastomal hernia (n=9)	Partial necrosis (n=1)	Ileus (n=1)	Abscesses (n=2)	Ischemia (n=1)	Chi-Square	P-value
Open Hartman's procedure (n=3)	2 (4.3%)	1 (2.2%)	-	-	-	-	37.930	0.720
Low anterior resection and end colostomy (n=7)	5 (10.9%)	1 (2.2%)	-	1 (2.2%)	-	-		
Laparoscopic loop ileostomy (n=19)	15 (32.6%)	3 (6.5%)	-	-	1 (2.2%)	-		
Laparoscopic Loop end Colostomy (n=9)	6 (13.0%)	2 (4.3%)	-	-	-	1 (2.2%)		
Laparotomy (n=6)	3 (6.5%)	1 (2.2%)	1 (2.2%)	-	1 (2.2%)	-		
open anterior resection with end colostomy (n=1)	1 (2.2%)	-	-	-	-	-		
Semi-obstructing sigmoid adenocarcinoma (n=1)	1 (2.2%)	-	-	-	-	-		

## Discussion

Stapling devices are now widely used in colorectal resections; however, the incidence and clinical implications of technical issues related to circular staplers remain poorly understood [14]. Circular staplers play a crucial role in creating anastomoses during colorectal surgeries. Furthermore, powered stapling systems reduce the physical effort required by surgeons to activate the device, which may provide advantages in achieving successful anastomosis formation [15]. The purpose of this study was to determine the incidence of complications in traditional and EEA stoma procedures and to identify correlations between patient characteristics, surgery types, and complication rates.

In this study, the stoma type was predominantly traditional rather than EEA (80.4% versus 19.6%), highlighting a clear predominance of traditional stoma formation. Overall, complications were observed in 28.3% of patients, with a slightly higher proportion in the EEA compared to the traditional group (33.3% versus 27.0%). The most common complication was PSH (17.4%), which occurred more frequently in the traditional group than in the EEA group (18.9% versus 11.1%). Less frequent complications included partial necrosis (2.2%) and ischemia (2.2%), which were reported only in the EEA group, as well as ileus (2.2%) and abscesses (4.3%), which were reported only in the traditional group. PSH is regarded as the most frequent complication following stoma formation. Moreover, abscess formation was relatively uncommon, possibly reflecting skilled tissue handling during routine stoma creation [16]. This finding suggests that the surgical team's proficiency in performing traditional stoma creation may contribute to a lower incidence of abscesses, emphasizing the crucial role of surgical technique in minimizing complications. Ultimately, the observed differences in complication rates between the two groups underscore the importance of surgical expertise in stoma formation.

In this study, the prevalence of PSH was lower in the EEA group. The findings regarding EEA-related complications align with previous studies, which have shown that stapler-based techniques may induce ischemia due to inadequate vascularization at the stoma site, leading to necrosis formation [17, 18]. This complication can significantly affect patient recovery and may require additional interventions, underscoring the importance of meticulous surgical technique. The increased incidence of necrosis in the EEA group can likely be attributed to the mechanism of tissue approximation. The EEA method depends on the use of a stapler, which in some cases may not achieve optimal tissue approximation, particularly in patients with thick abdominal walls or poor vascularity. A systematic review conducted by Martín-Arévalo et al. reported that although the use of circular staplers is effective, it carries a higher risk of complications, especially in patients with poorly vascularized tissue or those at high risk for ischemia and necrosis [19].

In contrast, the conventional method allows the surgeon to manually adjust tissue approximation, providing greater control over fascial tension and blood supply to the stoma site. Consequently, this technique ensures better preservation of vascularity and reduces the incidence of necrosis and other ischemic complications. A systematic review demonstrated that the manual approach is associated with fewer necrosis-related complications, which may be attributed to more precise tissue handling and vascular control [20-22]. This finding contrasts sharply with the EEA method, which may occasionally compromise blood circulation to the stoma, resulting in complications such as necrosis [19]. Another meta-analysis confirmed that manual stoma creation techniques are associated with fewer complications than stapler-based methods [16]. The results of the present study support these findings, suggesting that the conventional method can yield better outcomes in some instances. Although the EEA technique is faster and more standardized, it was associated with a higher incidence of complications, particularly necrosis, ischemia, and PSH. Given the smaller sample size in the stapler group (n=9) compared to the traditional group (n=37), results of this study should be interpreted with caution, as the statistical power is limited and may not reflect true clinical equivalence.

Along with surgical technique, patient characteristics, including BMI, age, and gender, are relevant to the occurrence of stoma-related complications. In this study, no significant associations were found between demographic characteristics (age, gender, and BMI) and the occurrence of complications (all p > 0.05). Complications were observed across all age groups, except in patients younger than 25 years, who reported no complications. PSHs were most frequent in the 51-75-year age group (8.7%). Both males and females experienced complications with a similar distribution (p = 0.822). Regarding BMI, patients with BMI >25 showed a higher proportion of complications, especially PSHs (13.0%) and other events, such as partial necrosis (2.2%) and ileus (2.2%). However, patients with BMI ≤ 25 had fewer complications, including parastomal hernia (4.3%), abscesses (2.2%), and ischemia (2.2%). Although these trends suggest that overweight or obesity may increase the likelihood of complications, the differences were not statistically significant.

Although this study did not reveal any direct links between these factors (age, gender, and BMI) and complications, the current literature suggests that elevated BMI and advanced age may be risk factors for the development of complications. Studies have concluded that patients with a higher BMI are more likely to develop PSHs due to high levels of intra-abdominal pressure and the presence of excess tissue in the stoma area [22]. Obesity is a specific problem since it causes the abdominal wall to become overloaded, which may result in the development of a hernia. The high complication rates observed in the EEA group in this study may be attributed to the increased BMI of patients, as the stapler method does not guarantee perfect approximation of tissue and fascia closure. Conversely, the regular method is more flexible, which can be of special value in obese patients or those with complex abdominal anatomy. The same assumption, that obesity and older age contribute to elevated risks of PSH, was confirmed in a retrospective study whose authors advised an equivalent exercise of caution in selecting the surgical technique [17]. This finding is consistent with the present study, as the routine approach appeared to offer greater benefits in preventing PSHs among patients at increased risk. The manual stoma formation method, also applied in high-risk patients, causes the fewest complications related to the patient's BMI or age [20]. Moreover, these findings highlight the significance of surgical technique in influencing patient outcomes. Patients with challenging anatomical features may benefit more from the traditional method, which provides better control over tissue handling.

In the present study, complications were observed across different surgical techniques for stoma creation, though no statistically significant differences were detected between groups. The highest complication rates were recorded following laparoscopic loop ileostomy and laparoscopic loop end colostomy, with PSH emerging as the most common adverse event. This finding is consistent with previous literature, where PSH is repeatedly identified as the most frequent long-term complication after stoma formation, with incidence rates ranging between 30% and 50%, depending on the surgical approach and follow-up duration [7, 23]. Interestingly, rare complications, such as partial necrosis (2.2%) and ischemia (2.2%), were observed following laparotomy and laparoscopic loop end colostomy, respectively. Similar findings have been reported in observational studies, where early complications, such as necrosis or ischemia, are documented in up to 5% of cases; however, their occurrence is strongly associated with technical factors, including blood supply and stoma site tension [24, 25]. Notably, no complications were observed in patients undergoing open anterior resection with end colostomy and surgery for semi-obstructing sigmoid adenocarcinoma in the present series. While this could reflect genuine differences in risk profiles, the absence of complications in these subgroups is more likely related to smaller patient numbers and limited follow-up. Previous studies emphasize that complication rates are highly dependent on patient characteristics, surgical technique, and follow-up duration, making small subgroup analyses difficult to interpret [26, 27]. Overall, although laparoscopic procedures showed a trend toward higher variability in complications, these differences were not statistically significant. This finding aligns with broader evidence suggesting that laparoscopic and open stoma formation have comparable complication profiles, though technical nuances and surgeon expertise may influence specific risks [23].

These findings have meaningful clinical relevance. Although the traditional method is more common for creating a stoma, this research suggests that surgeons should consider risk factors such as obesity and advanced age when determining the appropriate technique for stoma formation. Manual adjustment of tissue approximation and maintenance of proper vascularity are significant considerations in minimizing complications such as necrosis and PSH. From a cost perspective, traditional cruciate fascial incision remains more economical than stapler-assisted trephination, as stapler devices add material expense without demonstrable reduction in complication rates. This aligns with published findings reporting higher procedural costs for circular staplers [16]

This study has several limitations. First, the relatively small sample size (46 patients) reduces statistical power and limits the ability to detect subtle differences in complication rates between the two techniques. Second, being a single-center, hospital-based retrospective study, the findings may not be generalizable to other populations or healthcare settings. Third, variability in surgeon experience and technical preferences may have influenced outcomes, but this factor was not controlled for in the study design. Additionally, the retrospective nature of the study limited the ability to standardize definitions and timing of complication assessment, and some complications may have been underreported if they were managed outside the hospital's follow-up system. Given the smaller sample size in the stapler group compared to the traditional group, results should be interpreted with caution. Finally, the short follow-up period may have underestimated the true incidence of long-term complications such as parastomal hernia.

## Conclusions

The overall incidence of complications did not differ between circular stapler (EEA) and traditional cruciate fascial incision methods for stoma creation. While parastomal hernias were more common in the traditional group, ischemia and necrosis were observed only in the EEA group, and ileus and abscesses occurred only with the traditional method. Each method has distinct complication profiles, but neither demonstrated clear superiority. Larger, prospective, multicenter studies with longer follow-up are needed to more definitively evaluate the safety, complication rates, and long-term outcomes of these techniques and to guide surgical decision-making in stoma formation. Future prospective multicentre studies with standardized trephine diameter and predefined PSH imaging criteria are warranted.

## References

[1] Chen MZ, Gilmore A (2021). Short-term outcomes of parastomal hernia prophylaxis with Stapled Mesh stomA Reinforcement Technique (SMART) in permanent stomas. ANZ J Surg.

[2] Aquina CT, Iannuzzi JC, Probst CP, Kelly KN, Noyes K, Fleming FJ, Monson JR (2014). Parastomal hernia: a growing problem with new solutions. Dig Surg.

[3] Helgstrand F, Henriksen NA (2022). Outcomes of parastomal hernia repair after national centralization. Br J Surg.

[4] Mäkelä JT, Niskasaari M (2006). Stoma care problems after stoma surgery in Northern Finland. Scand J Surg.

[5] Kald A, Juul KN, Hjortsvang H, Sjödahl RI (2008). Quality of life is impaired in patients with peristomal bulging of a sigmoid colostomy. Scand J Gastroenterol.

[6] Carne PW, Robertson GM, Frizelle FA (2003). Parastomal hernia. Br J Surg.

[7] Liu L, Zheng L, Zhang M, Hu J, Lu Y, Wang D (2022). Incidence and risk factors for parastomal hernia with a permanent colostomy. J Surg Oncol.

[8] Ambe PC (2021). The safety of surgical technique for ileostomy and colostomy in preventing parastomal hernias: an in vitro experimental simulation study. Patient Saf Surg.

[9] Correa Marinez A, Bock D, Erestam S (2021). Methods of colostomy construction: no effect on parastomal hernia rate: results from stoma-const-a randomized controlled trial. Ann Surg.

[10] Koltun L, Benyamin N, Sayfan J (2000). Abdominal stoma fashioned by a used circular stapler. Dig Surg.

[11] Christakis C, Chatzidimitrou C, Kontos N, Papadopoulou S, Karanikas M (2004). Use of intraluminal stapler device for creation of a permanent colostomy. Tech Coloproctol.

[12] Kazi M, Desouza A, Vispute T, Nashikkar C, Saklani A (2023). Use of circular staplers for the creation of abdominal apertures for end colostomies: phase I study. Br J Surg.

[13] Cottam J, Richards K, Hasted A, Blackman A (2007). Results of a nationwide prospective audit of stoma complications within 3 weeks of surgery. Colorectal Dis.

[14] Offodile AC 2nd, Feingold DL, Nasar A, Whelan RL, Arnell TD (2010). High incidence of technical errors involving the EEA circular stapler: a single institution experience. J Am Coll Surg.

[15] Herzig DO, Ogilvie JW, Chudzinski A (2020). Assessment of a circular powered stapler for creation of anastomosis in left-sided colorectal surgery: a prospective cohort study. Int J Surg.

[16] Bai J, Zhao Y, Liang H, Li J, Zhang C (2022). Indirect comparison between powered and manual circular staplers for left-sided colorectal anastomoses: clinical and economic outcomes in China. Cost Eff Resour Alloc.

[17] Benedek Z, Kocsis L, Bauer O (2022). Stoma-related complications: a single-center experience and literature review. J Interdiscip Med.

[18] Parini D, Bondurri A, Ferrara F (2023). Surgical management of ostomy complications: a MISSTO-WSES mapping review. World J Emerg Surg.

[19] Martín-Arévalo J, Pla-Martí V, Huntley D (2024). Two-row, three-row or powered circular stapler, which to choose when performing colorectal anastomosis? A systematic review and meta-analysis. Int J Colorectal Dis.

[20] Kumano K, Kitaguchi D, Owada Y (2023). A comparative study of stoma-related complications from diverting loop ileostomy or colostomy after colorectal surgery. Langenbecks Arch Surg.

[21] Sadiq KO, Lakshminarayanan S, Ruiz Cota P, Marquez Castillo E (2025). Higher BMI increases risk of stoma-site incisional hernia and other complications following diverting loop ileostomy and reversal: a systematic review and meta-analysis. Surg Endosc.

[22] Berger D (2023). Perspectives of prevention and treatment of parastomal hernia-what do we really know and where should we go?. Minim Invasive Surg.

[23] López-Cano M, Teresa Quiles M, Antonio Pereira J, Armengol-Carrasco M, Arbós Vía MA (2017). Complex abdominal wall hernia repair in contaminated surgical fields: factors affecting the choice of prosthesis. Am Surg.

[24] Shabbir J, Britton DC (2010). Stoma complications: a literature overview. Colorectal Dis.

[25] Krishnamurty DM, Blatnik J, Mutch M (2017). Stoma complications. Clin Colon Rectal Surg.

[26] Manole TE, Daniel I, Alexandra B, Dan PN, Andronic O (2023). Risk factors for the development of parastomal hernia: a narrative review. Saudi J Med Med Sci.

[27] Bişgin T, AyİK C, Cenan D, Manoğlu B, Özden D, Sökmen S (2023). The risk factors for parastomal hernia development: a 8-year retrospective study in colorectal surgery. J Basic Clin Health Sci.

